# The response to single-gene duplication implicates translation as a key vulnerability in aneuploid yeast

**DOI:** 10.1371/journal.pgen.1011454

**Published:** 2024-10-25

**Authors:** H. Auguste Dutcher, James Hose, Hollis Howe, Julie Rojas, Audrey P. Gasch

**Affiliations:** 1 Center for Genomic Science Innovation, University of Wisconsin-Madison, Madison, Wisconsin, United States of America; 2 Laboratory of Genetics, University of Wisconsin-Madison, Madison, Wisconsin, United States of America; University of Georgia, UNITED STATES OF AMERICA

## Abstract

Aneuploidy produces myriad consequences in health and disease, yet models of the deleterious effects of chromosome amplification are still widely debated. To distinguish the molecular determinants of aneuploidy stress, we measured the effects of duplicating individual genes in cells with different chromosome duplications, in wild-type cells (*SSD1*+) and cells sensitized to aneuploidy by deletion of RNA-binding protein Ssd1 (*ssd1Δ*). We identified gene duplications that are nearly neutral in wild-type euploid cells but significantly deleterious in euploids lacking *SSD1* or in *SSD1+* aneuploid cells with different chromosome duplications. Several of the most deleterious genes are linked to translation. In contrast, duplication of other genes benefits multiple *ssd1Δ* aneuploids over controls, and this group is enriched for translational effectors. Furthermore, both wild-type and especially *ssd1Δ* aneuploids with different chromosome amplifications show increased sensitivity to translational inhibitor nourseothricin. We used comparative modeling of aneuploid growth defects, based on the cumulative fitness costs measured for single-gene duplication. Our results present a model in which the deleterious effects of aneuploidy emerge from an interaction between the cumulative burden of many amplified genes on a chromosome and a subset of duplicated genes that become toxic in that context. These findings provide a perspective on the dual impact of individual genes and overall genomic burden, offering new avenues for understanding aneuploidy and its cellular consequences.

## Author summary

Aneuploidy, when cells carry an imbalanced set of chromosomes, creates cellular defects relative to euploids, but the molecular causes of these deleterious effects are not known. One possibility is that having an entire extra chromosome of gene products is inherently stressful to cells; another is that extra copies of specific individual genes are highly toxic. In this study, we investigate aneuploidy stress in yeast by duplicating individual genes in euploids and aneuploids in a wild yeast model, and identify that some genes with little effect in one context are uniquely deleterious in the other. We use modeling to show that context-dependent gene toxicity helps explain aneuploidy defects and that cells with extra chromosomes may have a vulnerability linked to translation.

## Introduction

Aneuploidy, the state of having an abnormal number of one or more chromosomes, is highly relevant to human health: it underlies embryonic inviability and trisomy syndromes, is associated with aging, and is a well-known feature of malignant cancers [1–3]. Debates continue over the mechanisms driving the deleterious consequences of aneuploidy, specifically the negative effects of chromosome duplication. Models range from focusing on the increased generic burden of extra DNA and encoded protein [4,5], to stoichiometric imbalance of dosage-sensitive proteins [6–8], to amplification of specific highly toxic gene products [9,10]. However, distinguishing between the deleterious effects of specific genes versus the collective burden of many genes is a significant experimental challenge, especially in mammalian systems. Thus, despite the relevance and prevalence of aneuploidy, we still lack a comprehensive understanding of the mechanisms behind the deleterious consequences of chromosome amplification.

Research in the yeast model organism, *Saccharomyces cerevisiae*, has expanded our understanding of the cellular impacts of aneuploidy and led to the discovery of key genetic vulnerabilities. Pioneering studies using the highly aneuploidy-sensitized laboratory strain W303 unveiled a range of defects in cells with extra chromosomes. These include metabolic changes, cellular stress, defects in cell-cycle progression, and signs of protein aggregation and protein mismanagement known as proteostasis stress [4,11–15]. However, work from our lab and others has shown that the W303 is an outlier in its extreme sensitivity to chromosome duplication. In contrast to the W303 strain, aneuploidy is not uncommon in non-laboratory strains of yeast and found in ~20% of strains distributed across the *S*. *cerevisiae* phylogeny [16,17]. Strains with extra chromosomes typically grow substantially better than W303, though often with slower growth rates than isogenic euploid cells, in both wild yeast strains and a different lab strain derived from S288c [16–20]. We previously showed that the W303 strain’s extreme sensitivity to chromosome duplication is due to a hypomorphic allele of the RNA-binding protein Ssd1 [21]. Deletion of *SSD1* from wild strains recapitulates many of the aneuploidy phenotypes reported in W303, including signs of proteostasis stress [21]. Ssd1 is thus a key mediator of aneuploidy tolerance, though its precise role in this modulation has remained elusive.

Ssd1, orthologous to human Dis3L2 but lacking nuclease activity, binds several hundred mRNAs via sequence motifs often found in the 5’ UTR [21–26]. Bound mRNAs are enriched for a variety of encoded functions, including proteins localizing to the cell wall and proteins involved in cell cycle regulation, RNA metabolism, sterol transport, and a diverse collection of seemingly unrelated functions [21,22,24]. Although molecular details are sparse, Ssd1 plays a role in translation and localizing mRNAs to processing bodies and the cellular periphery, particularly for encoded proteins localized to the cell wall or nascent bud [22,23,26–31]. Recent findings from our laboratory demonstrated that deleting *SSD1* sensitizes a wild strain to most chromosome duplications to varying degrees [32], indicating that the role of Ssd1 in aneuploidy tolerance is not limited to a specific chromosome. Thus, understanding the role of Ssd1 in tolerating chromosome amplification is a potent tool for investigating the molecular determinants of aneuploidy tolerance more broadly.

Separate from the role of Ssd1, the molecular determinants behind aneuploidy toxicity in functional cells are still widely debated. Work from *S*. *cerevisiae* transformed with yeast artificial chromosomes (YACs) containing human DNA implicated gene products—not merely the presence of extra DNA—as the cause of aneuploidy-related defects [4,33]. This fostered the idea that proteostasis stress resulting from many amplified genes drives aneuploidy toxicity in sensitized cells [11,34]. One hypothesis is that simply having too much protein, regardless of what the proteins are, produces proteostasis stress. But another is that the over-abundance of specific proteins causes stoichiometric imbalance. Focus has landed on proteins in multi-subunit complexes and those with many protein-protein interactions, since these may be most affected by stoichiometry [7,35,36]. Both cases could cause a range of effects including protein aggregation [37], defects with protein quality control [11], and resulting osmotic challenges that disrupt endocytosis and other processes [38]. An alternate theory is that amplification of specific genes encoded on some chromosomes could pose particular problems. Given that whole chromosome amplification inherently increases the copy number of many genes simultaneously, distinguishing the effects of specific genes from generalized burden remains a complex task.

In this study, we leveraged Ssd1 to help distinguish between these possibilities. We used a yeast gene expression library to identify genes that are deleterious when duplicated in isolation, in euploid and aneuploid cells with and without *SSD1* (referred to as *SSD1+* and *ssd1Δ*, respectively). Assaying euploids and aneuploids enabled us to identify differential effects of gene duplication in both contexts. This approach identified shared susceptibilities in *ssd1Δ* euploid cells and *SSD1+* aneuploids, revealing that *SSD1* deletion in euploids partly phenocopies the stress of aneuploidy in wild-type cells. We propose that the stress of chromosome duplication results from an interaction between the generalized burden of chromosome amplification with specific genes whose duplication is largely neutral in euploid cells but becomes problematic in the aneuploid context. Our study demonstrates the value of leveraging genetic vulnerabilities to deepen our understanding of the cellular state of aneuploidy and its implications for human health.

## Results

We set out to investigate the response of *ssd1Δ* cells to chromosome duplication. We leveraged a recent study from our lab that generated a panel of haploid *Saccharomyces cerevisiae* strains, each carrying an extra copy of single chromosomes [32]. This strain panel was engineered in a haploid derivative of oak-soil strain YPS1009 and included all chromosomes except chromosome VI (Chr6) that is toxic [4,39]. That work also generated a comparable panel in YPS1009 cells lacking *SSD1*, excluding Chr16 duplication that was unculturable in the absence of Ssd1. We previously observed that many of the aneuploid strains showed reduced growth rates in the absence of *SSD1*, although the models of Ssd1 involvement were not pursued further [32]. Here, we specifically analyzed the growth rates of *ssd1Δ* aneuploids to better understand how Ssd1 is involved in aneuploidy tolerance.

At the outset, we envisioned two possibilities for Ssd1’s role. One possibility is that Ssd1 manages the stress of chromosome duplication seen in wild-type cells, such that deletion of *SSD1* exacerbates that stress. If so, the degree of Ssd1 dependence for each chromosome duplication would be proportionate to the fitness cost of that chromosome duplication in the wild type. This should produce a linear relationship between Ssd1 dependence and inherent chromosome fitness costs. An alternative possibility is that deletion of *SSD1* introduces entirely new sensitivities that are not relevant in the *SSD1+* aneuploids. In this scenario, the degree of Ssd1 dependence may be uncorrelated with chromosome toxicity in the wild-type cells.

**Fig 1:**
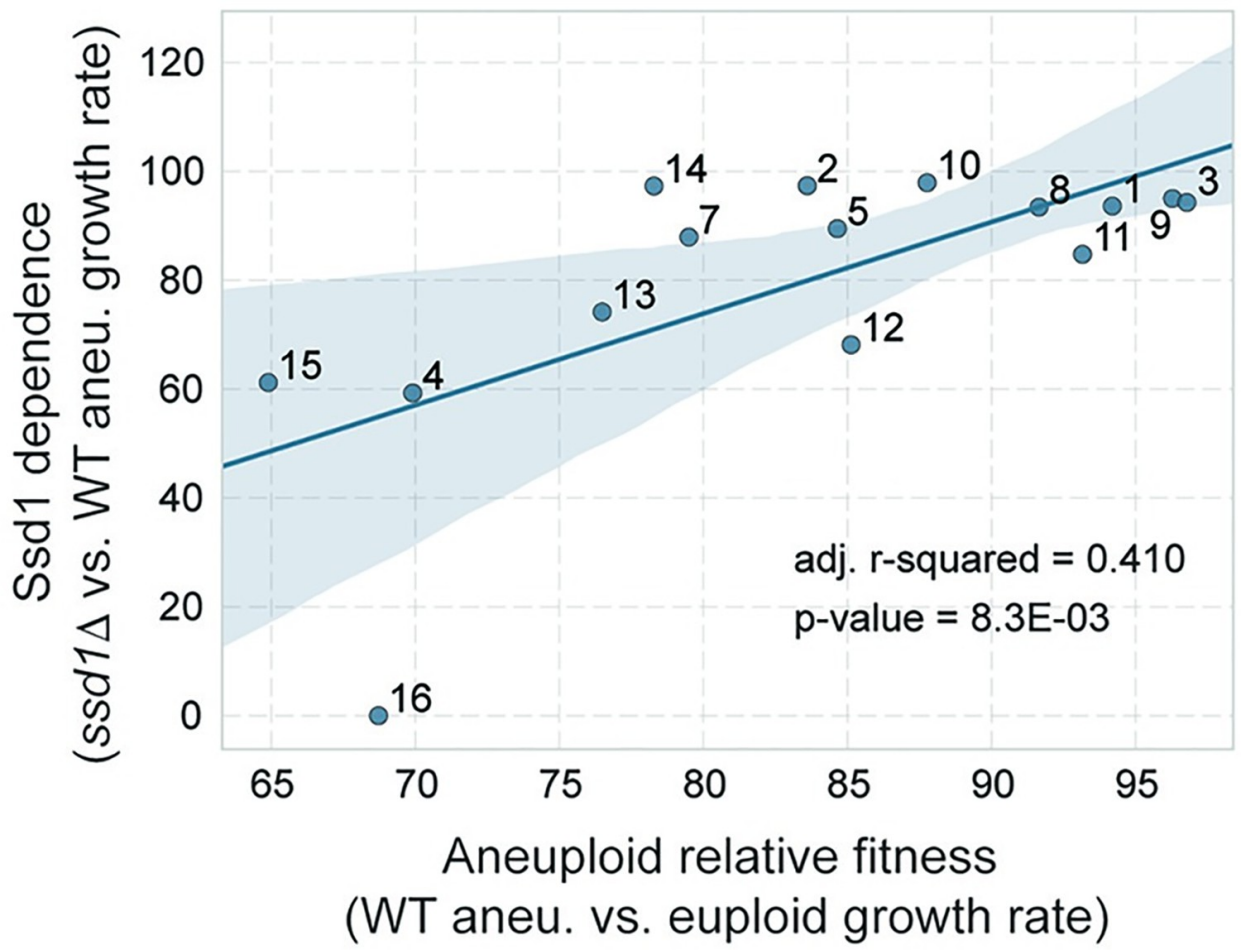
Ssd1 dependence is modestly correlated with aneuploidy toxicity in the *SSD1+* wild-type background. The y-axis shows the Ssd1 dependence of each chromosome duplication, taken as the average (n = 4) growth rate of the *ssd1Δ* aneuploid versus the average (n = 4) wild-type aneuploid. The x-axis shows the relative fitness of each aneuploid strain, taken as the average (n = 4) growth rate of wild-type aneuploid versus the corresponding average for the euploid. Growth rates were reported in [32]. Points are labeled to identify the amplified chromosome. The line shows ordinary least squares regression; shaded area denotes the 95% confidence interval (see Methods).

To distinguish these possibilities, we plotted Ssd1 dependence against the fitness cost of each chromosome. We defined the Ssd1 dependence of each chromosome as the relative growth rate of the *ssd1Δ* aneuploid versus the wild-type aneuploid with the same chromosome duplication. We then plotted this against the relative growth rate of each wild-type aneuploid versus the euploid control (**Fig 1**). We found a modest yet significant correlation between Ssd1 dependence and chromosome fitness cost (R^2^ = 0.41, p = 8.3E-03). However, several chromosome amplifications were substantially more or less dependent on Ssd1 than predicted from the chromosome cost in the wild type. For example, tolerance of Chr12 duplication was very dependent on Ssd1, surpassed only by Chr16 duplication that is unculturable in the *ssd1Δ* strain. Conversely, Chr2, Chr14, and Chr10 amplifications were proportionately less dependent on Ssd1 than expected. Together these findings imply that Ssd1 has a generalizable role in coping with aneuploidy, but with some chromosome-specific effects. Thus, Ssd1 deletion is a useful tool to exacerbate and interrogate the stress of chromosome duplication.

### A gene expression library reveals costs of single gene duplication

**Fig 2:**
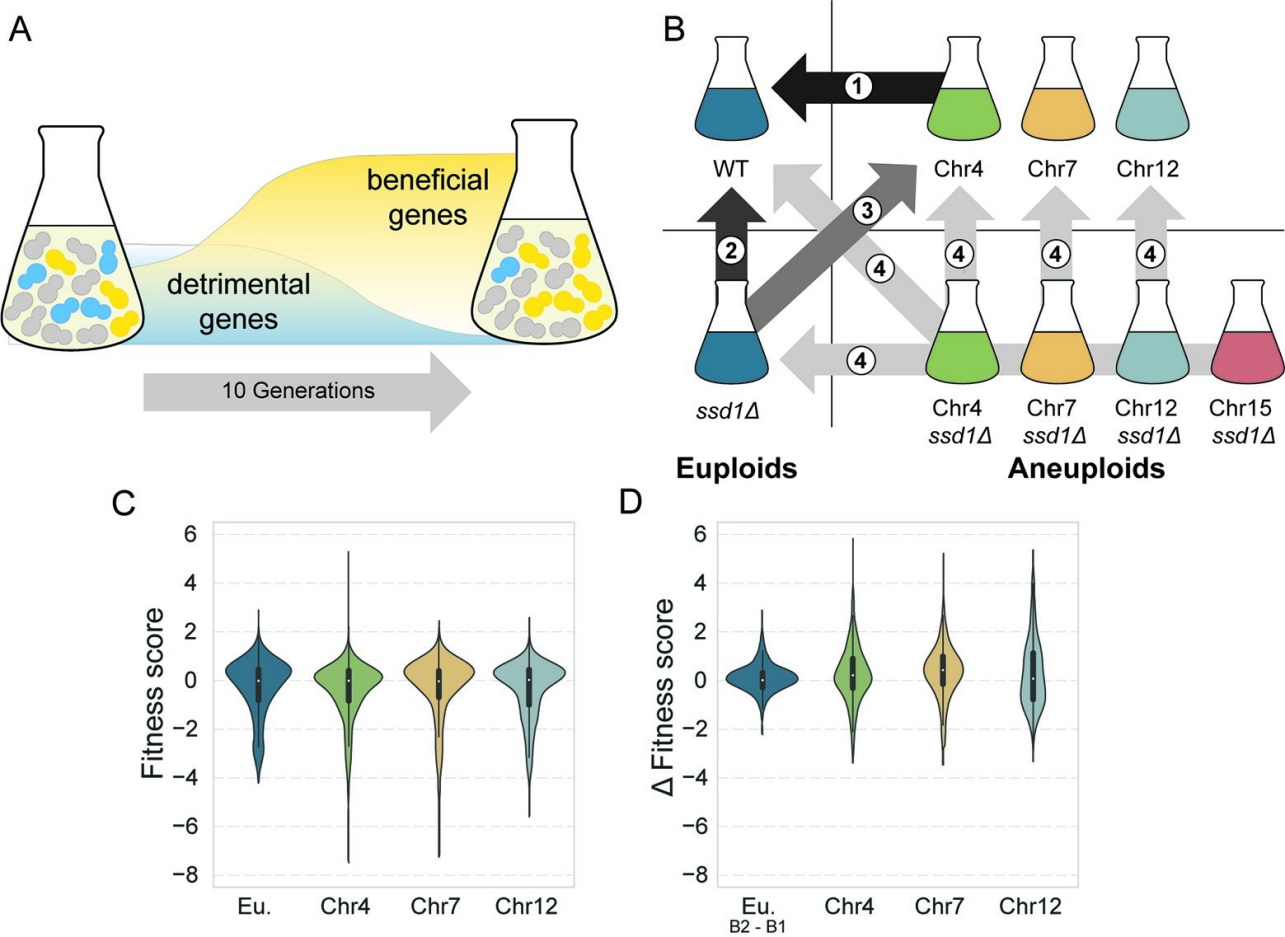
Distribution of fitness costs of gene duplication are similar in aneuploids and euploids. A) Schematic of the approach, see text for details. B) Library competition experiments were performed in strains indicated in the diagram. Specific comparisons (indicated by a number) were made to compare: (1) Wild-type aneuploids versus wild-type euploid; (2) *ssd1Δ* euploid versus wild-type euploid; (3) *ssd1Δ* euploid versus wild-type aneuploids, used for modeling; (4) *ssd1Δ* aneuploids versus wild-type euploid, *ssd1Δ* euploid, and corresponding wild-type aneuploid when available. C) Distribution of fitness scores (log_2_(fold change) of normalized barcode counts) for all measured genes in euploid (Eu.) and aneuploids with the denoted chromosome amplification. D) Distribution of pairwise differences in log_2_ fitness scores between each wild-type aneuploid and batch-paired euploids (Comparison 1 in B), for 2,403 detrimental genes identified in the euploid wild-type [32]. Eu_B2-B1_ represents a control distribution comparing fitness scores in two batches (B1 and B2) of euploid replicates. https://commons.wikimedia.org/wiki/File:Erlenmeyer_Flask_%2822792%29_-_The_Noun_Project.svg.

Previous studies strongly suggested that aneuploidy toxicity is dependent on genes encoded on the amplified chromosomes, since large DNA segments without coding potential are not especially toxic in yeast [4,33]. But whether aneuploidy toxicity, and Ssd1 dependence in particular, is due to only a few specific genes or the cumulative burden of many genes was unclear. To distinguish between models and study Ssd1’s role in aneuploidy tolerance, we measured the cost of individual gene duplications, using a barcoded plasmid library (**Fig 2A**). The MoBY 1.0 gene library comprises ~5,000 open reading frames along with their native upstream and downstream sequences, cloned onto a barcoded centromeric (CEN) plasmid [40]. We transformed the library into wild-type and *ssd1Δ* euploid strains as well as select wild-type and *ssd1Δ* aneuploids with duplications of Chr4, Chr7, Chr12, and Chr15 (the Chr15 wild-type aneuploid was omitted due to poor library coverage). These strains were chosen because they present a range of growth defects and varying degrees of Ssd1 dependence. Each copy of the duplicated chromosomes carried a different selectable marker, allowing both copies of the chromosome to be maintained throughout the experiment. Each pooled library was grown for 10 generations (with the exception of the YPS1009 Chr4 *ssd1Δ* strain, which was grown for only 5 generations due to its extreme fitness defect), in at least 3 biological replicates (see Methods). An aliquot of the library was collected before and after competitive growth, and the relative abundance of each plasmid in each sample was scored by sequencing the plasmid barcodes (see Methods). Barcodes that decreased in abundance during competitive growth correspond to deleterious gene duplicates, whereas barcodes that increased in abundance represent genes whose duplication is advantageous. To quantify fitness effects, we calculated a fitness score for each gene duplication, defined as the log_2_(fold change) of normalized barcode counts after competitive growth (see Methods). We applied several types of comparisons to understand the impact of aneuploidy in *SSD1+* and *ssd1Δ* cells (**Fig 2B**). Data for the euploid YPS1009 were first reported in a parallel paper from our lab [32].

As reported in this parallel paper, duplication of 2,403 genes produced a significant fitness effect in the euploid strain at a false discovery rate (FDR) < 0.05 [32]. Re-analysis here showed that beneficial gene duplicates were enriched for essential genes (p = 9.1E-08, hypergeometric test). Genes whose duplication was detrimental were not enriched for any gene ontology terms; however, we previously noted that deleterious genes tended to be longer and encode proteins with more disorder [32], consistent with other reports [41]. There was no enrichment for proteins in multi-subunit complexes and no difference in the number of protein interactions for deleterious genes compared to neutral genes. None of the chromosomes showed a significant overrepresentation of detrimental genes that passed FDR significance (FDR > 0.05, hypergeometric test with Benjamini and Hochberg correction).

### Aneuploid strains are not universally more sensitive to gene duplications

Here we focus on the effects of single-gene duplication in aneuploid strains. We initially hypothesized that aneuploids may be more sensitive to the gene duplication library, since they already carry a burden of extra gene content. If aneuploids were more sensitive to gene duplication, we would expect more deleterious genes in aneuploid strains and/or more severe fitness defects upon duplication. On a global scale, this was not the case: the proportion of detrimental genes was comparable between aneuploids and batch-paired euploids (see S1 Table), albeit with a marginally higher proportion of detrimental genes in aneuploids as seen in comparing the distributions (**Fig 2C**). This could not be explained by data normalization issues, since we confirmed that genes scored as neutral in the library experiment had no significant effect on growth rate when strains were grown in isolation (S1 Fig). Although there was a longer tail of genes with stronger negative effects in the aneuploid strains (**Fig 2D**), the bulk of the distribution was similar to euploids. We conclude that the aneuploid strains are not sensitized to over-expression of all genes, but they are overly sensitive to a subset of duplicated genes.

To investigate further, we identified 335 genes that were deleterious in one or more aneuploids (FDR < 0.05) with a fitness score at least 1.5 in log_2_ space more deleterious than batch-paired euploid measurements (Comparison 1 in Fig 2B, see Methods and S1 Data). Of these 335 deleterious gene duplicates, 58 were common to two or more aneuploid strains, suggesting common effects across aneuploid strains. There was no enrichment for any gene ontology terms. However, we noticed that 8 of the common detrimental genes are naturally encoded adjacent to a centromere, such that the centromere was cloned onto the plasmid along with the gene. Dicentric plasmids are known to be unstable in yeast [42], likely because spindle attachment to both centromeres results in DNA breakage [43]. The number of especially deleterious plasmids that carried a cloned centromere was higher than expected by chance (p = 5.5E-09, hypergeometric test). This raised the possibility that plasmids with multiple centromeres may be especially unstable in aneuploid strains relative to euploids.

In contrast, we noticed that some genes scored as deleterious in the euploid were much less deleterious in aneuploids (**Fig 2D**). We identified 360 genes that were deleterious in the euploid strain (FDR < 0.05) but at least 1.5 in log_2_ space less so in one or more wild-type aneuploids (Comparison 1 in Fig 2B and S1 Data). 153 of these genes were common to two or more aneuploid strains, again suggesting common effects independent of which chromosome is duplicated. Intriguingly, this group was enriched for genes involved in the mitotic spindle (p = 9.1E-05, hypergeometric test). Previous work by Storchova *et al*. [44] showed that a subset of genes affecting the mitotic spindle are essential in tetraploid, but not diploid, yeast. The authors proposed that increased chromosome content may alter requirements for spindle geometry for proper chromosome management. Our results hint that aneuploid cells with extra chromosomes may benefit from increased abundance of spindle components. In sum, our results suggest that aneuploid strains are not generally more sensitive to further gene duplication but that a subset of genes are more or less toxic in aneuploids.

### Specific gene duplications are especially toxic in *ssd1Δ* euploids and explain a portion of Ssd1 aneuploidy dependence

**Fig 3:**
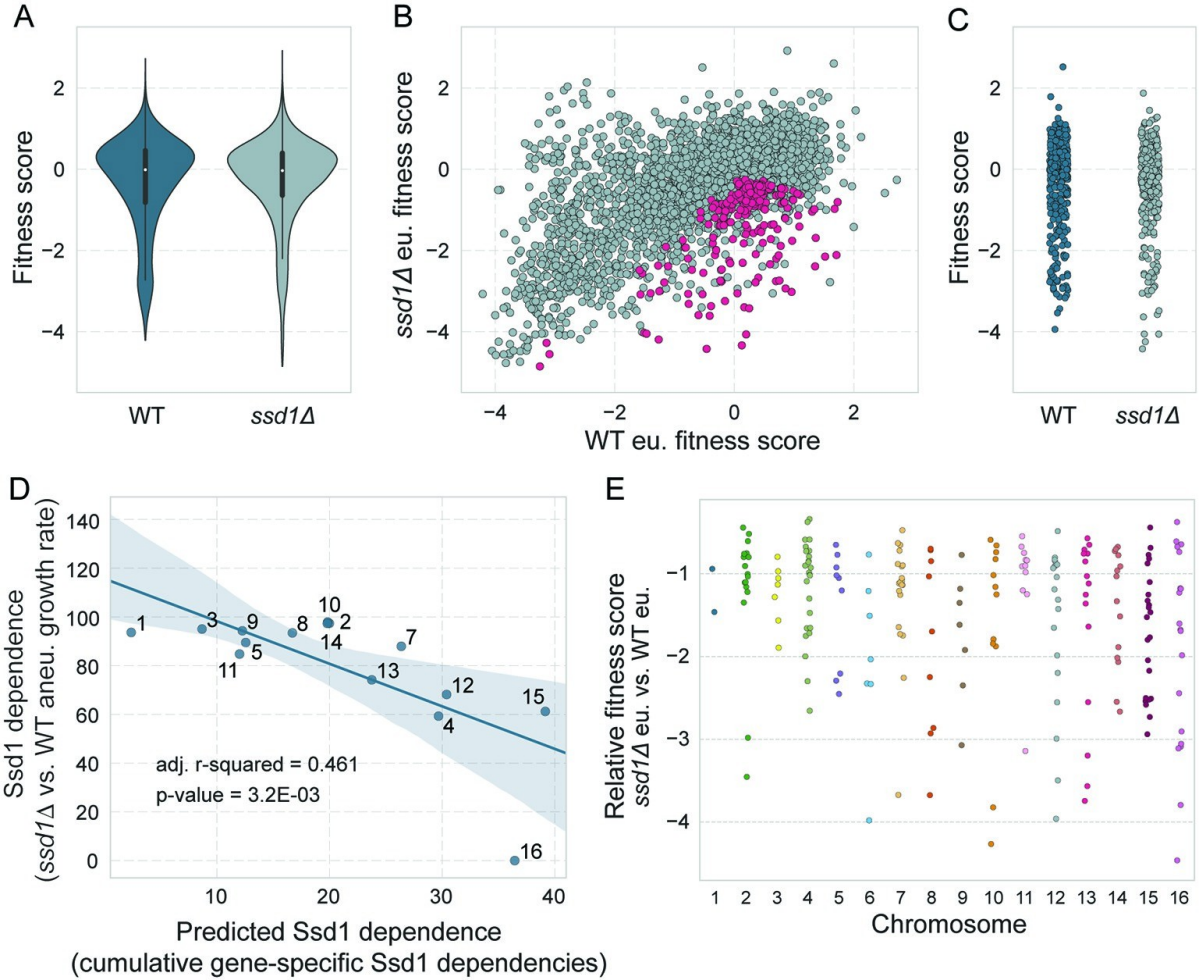
A subset of genes are more detrimental in *ssd1Δ* euploid cells relative to wild type. A) Distributions of fitness scores for all 4,462 measured genes in wild-type euploid and *ssd1Δ* euploid strains. B) Scatterplot of replicate-averaged fitness scores for all measured genes in *ssd1Δ* euploid (y-axis) and wild-type euploid (x-axis); 218 genes significantly more deleterious in *ssd1Δ* euploid cells are shown in pink. C) Fitness scores for 336 genes encoding mRNAs bound by Ssd1 (see Methods) in wild-type (blue) and *ssd1Δ* (light blue) euploid strains. D) Ssd1 dependence as shown in Fig 1 (y-axis) plotted against the predicted Ssd1 dependence, calculated as the sum of log_2_ gene-specific Ssd1 dependence values as described in the text (x-axis), over the subset of 218 genes whose duplication is toxic to *ssd1Δ* cells. E) Ssd1-dependent fitness scores for the 218 genes analyzed in (D), plotted according to encoding chromosome.

As shown above, *SSD1* deletion sensitizes cells to aneuploidy to varying degrees depending on which chromosome is amplified. Given Ssd1’s role in translational suppression, we wondered if *SSD1* deletion sensitizes cells to gene over-production in general. We therefore used the library to measure fitness costs of single-gene duplication in the *ssd1Δ* euploid. Overall, the distribution of fitness scores for gene duplications was not significantly different than the euploid wild type (p = 0.38, Wilcoxon rank-sum test, **Fig 3A**), indicating that *ssd1Δ* does not sensitize cells to all gene duplications. We directly compared *ssd1Δ* euploid versus wild-type euploid cells (Comparison 2 in Fig 2B) and identified 218 genes more detrimental in the *ssd1Δ* euploid (FDR < 0.05, **Fig 3B** and S1 Data). We were especially interested in identifying functional or biophysical properties of these genes; however, the group did not have striking functional enrichments, aside from 4 genes involved in ER to Golgi transport (p = 1.7E-03, hypergeometric test). We tested more than a dozen other transcript or protein features including abundance, structure or sequence composition, or other biophysical properties, but found nothing significant (see Methods). Furthermore, the group was not enriched for mRNAs bound by Ssd1 (p = 0.67, hypergeometric test) or that harbor the known Ssd1 binding motifs [22,24]. Reciprocally, genes encoding Ssd1-bound mRNAs were not more deleterious when duplicated in the absence of *SSD1* (**Fig 3C**). The only significant feature we found was that the length distribution of these 218 genes is shorter than all other genes in the dataset (p = 1.3E-04, Mann-Whitney U test). This is notable since genes whose duplication is deleterious in the wild type tend to be longer than neutral genes [32]. Thus, it is unclear why these 218 gene duplicates are more deleterious in *ssd1Δ* cells.

Nonetheless, we used these fitness scores to further understand models of Ssd1 dependence. In other recent work from our lab, we showed that nearly 70% of the variance in wild-type aneuploid growth rates can be explained by the cumulative fitness costs of genes duplicated on each chromosome [32]. Here we used a similar approach to explain Ssd1 dependence. We tested if the Ssd1 dependence for each chromosome duplication (as shown in Fig 1) could be explained by the cumulative Ssd1 dependencies of genes duplicated on that chromosome. We calculated Ssd1 dependence for each gene as the fitness cost for that gene duplication in the *ssd1Δ* strain versus the fitness cost for that gene duplication in the *SSD1+* euploid strain. We then calculated the per-chromosome Ssd1 dependence as the sum of relative log_2_ fitness scores over the 218 genes more deleterious in the mutant strain (see Methods). This simple model explained roughly half of the variance in Ssd1 aneuploidy dependence, with an adjusted R^2^ of 0.46 (**Fig 3D**, p-value = 3.2E-03). Omitting Chr16 from consideration increased the explanatory power to 51% (adj. R^2^ 0.51). Model performance decreased when only the top 10% of *ssd1Δ* -sensitive genes were used (36% of variance explained, S2 Fig). Thus, Ssd1 dependence for tolerating chromosome duplications can be partly explained by the cumulative burden of specific genes on each chromosome; however, this number is more than one or two genes per chromosome (**Fig 3E**).

### Many gene duplicates toxic in *ssd1Δ* euploids are also toxic to *SSD1+* aneuploids but not *SSD1+* euploids

**Fig 4:**
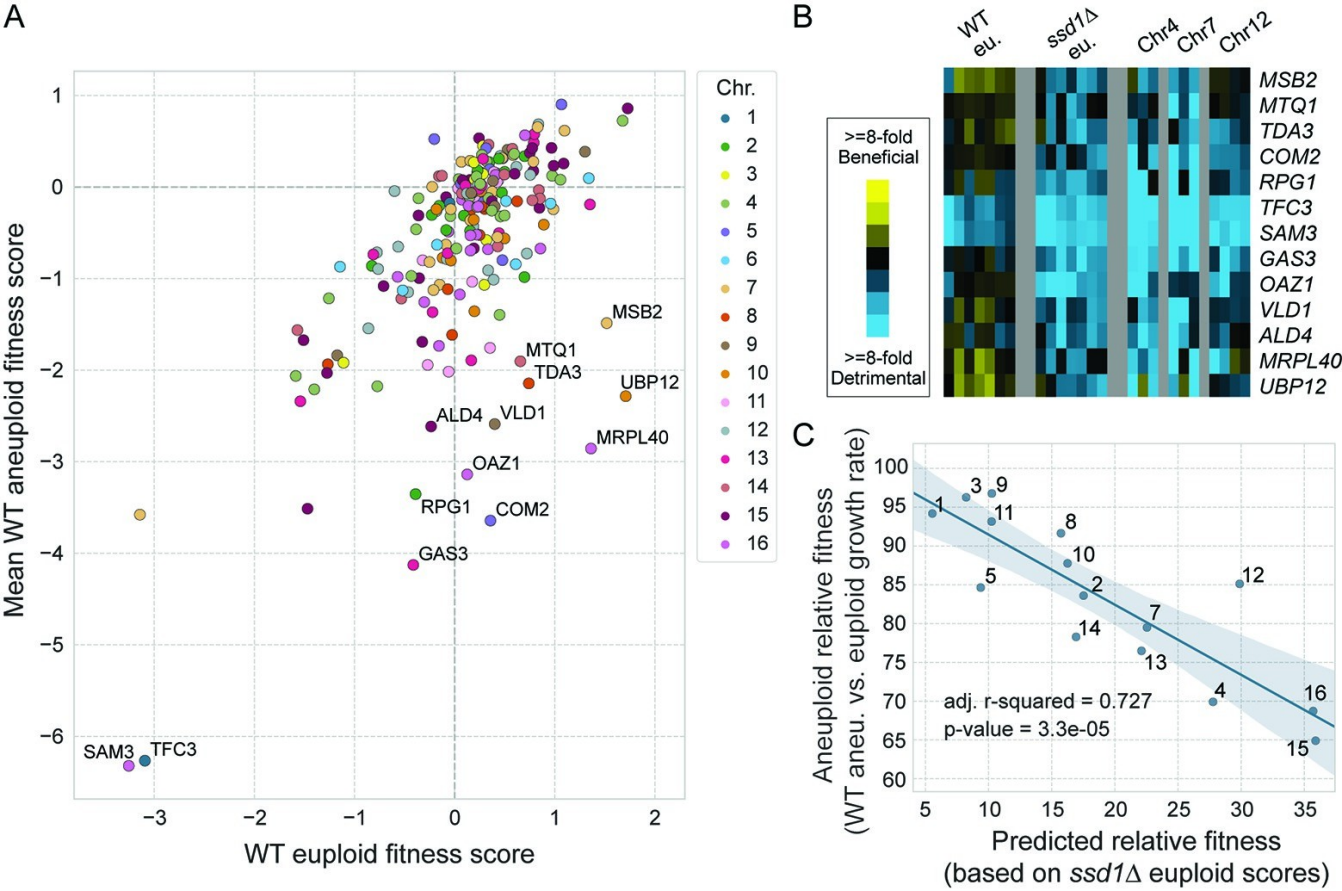
Many gene duplications toxic in *ssd1Δ* euploids are also toxic in *SSD1+* aneuploids. A) Average wild-type aneuploid fitness scores for each gene (y-axis) plotted against the corresponding fitness score measured in wild type euploid cells (x-axis), for the 218 genes in Fig 3B. Genes with especially deleterious effects in aneuploids relative to the euploid are labeled with gene name. B) Fitness scores for genes labeled in Fig 4A (rows) in biological replicates of each strain (columns), colored according to the key. C) The relative fitness of aneuploid strains (as in Fig 1) was plotted against the predicted relative fitness, taken as the sum of log_2_ fitness values for the 218 gene duplicates as measured in the *ssd1Δ* euploid strain (x-axis).

Ssd1 is required to tolerate specific gene duplications, although the reasons are unclear. We wondered if *SSD1+* aneuploids may be sensitive to the same set of genes. We explored this in several ways. First, we realized that genes more deleterious in one or more aneuploid strains (Comparison 1 in Fig 2B) are enriched for the 218 genes whose duplication is especially deleterious in *ssd1Δ* euploid cells (Comparison 2 in Fig 2B, p = 2.9e-06, hypergeometric test). Second, we interrogated the fitness scores of these 218 genes in wild-type aneuploid cells. We plotted the average fitness scores from the three wild type aneuploids against the euploid fitness scores for these 218 genes (**Fig 4A**). Indeed, 145 (67%) of these genes were more deleterious in aneuploids than in the euploid. One subset of genes was especially more toxic in aneuploids, indicated by their deviation from the linear trend (**Fig 4A**, labeled points). We confirmed that these genes were more deleterious in individual aneuploids compared to the euploid (**Fig 4B**). Thus, many of the gene duplications that are toxic in *ssd1Δ* euploids are also more deleterious in *SSD1+* aneuploids versus the euploid. This indicates that aneuploidy may be mimicking the cellular state of *ssd1Δ* euploid cells (see Discussion).

We wondered how well the cumulative cost of these 218 gene duplicates, identified by their sensitivity in the *ssd1Δ* euploid, could explain chromosome fitness costs in *SSD1+* aneuploids. We calculated the fitness cost of each chromosome duplication in *SSD1+* cells as the cumulative cost of these 218 gene duplicates, based on measurements from the wild-type euploid strain (see Methods). The calculated fitness costs did not agree with the measured costs in the *SSD1+* background (adjusted R^2^ = 0.07, p-value = 0.18). However, many of these genes are only deleterious when duplicated in the *ssd1Δ* euploid strain. This raised the possibility that the fitness costs of these gene duplicates in the *ssd1Δ* euploid was most relevant. We therefore modeled the fitness cost of chromosome duplication in *SSD1+* cells, but based on gene fitness costs measured in the *ssd1Δ* euploid strain. Remarkably, this model explained 73% of the variance in growth rates of the *SSD1+* aneuploids (**Fig 4C**). This important result indicates that chromosome duplication in an *SSD1+* background may mimic the liabilities of *SSD1* deletion in a euploid strain, sensitizing cells to the same subset of problematic gene duplications (see Discussion).

### Duplicated genes beneficial in *ssd1Δ* aneuploids implicate mechanisms of aneuploidy tolerance

**Fig 5:**
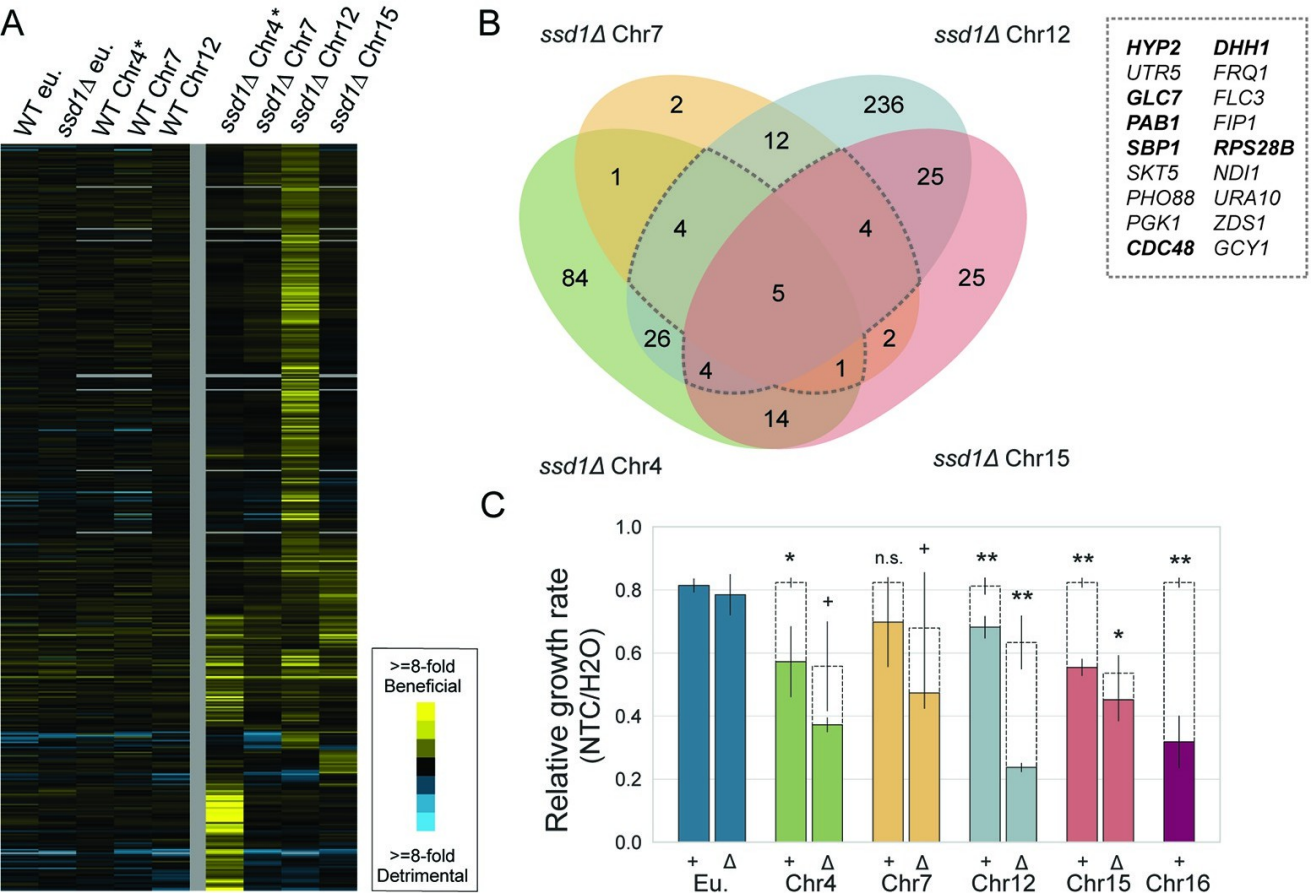
Gene duplications that benefit *ssd1Δ* aneuploids implicate translation. A) Hierarchically clustered fitness scores for 445 gene duplications (rows) beneficial in at least one aneuploid strain (columns), colored according to the key. Fitness scores represent the average of at least biological triplicate. (*) denotes scores measured after 5 generations instead of 10 generations, see text. B) Venn diagram of beneficial genes identified in *ssd1Δ* aneuploids. Dotted border identifies beneficial genes common to 3 or more *ssd1Δ* aneuploids. The inset lists 18 genes identified in 3 or more ssd1Δ aneuploids; bolded genes have known links to translation. C) Average and standard deviation of relative growth rates of the denoted strains grown in 1ug/mL nourseothricin (NTC) compared to a water control (n = 3). Measured growth rates are indicated as solid bars; expected growth rates based on the combined effect of NTC and underlying aneuploidy growth defect are shown as dashed bars (see Methods). (**) p-value < 0.01, (*) p < 0.05, (+) p < 0.1, one-tailed paired T-test.

Since *SSD1* deletion sensitizes strains to the stress of chromosome duplication, we wondered if genes beneficial in *ssd1Δ* aneuploids might complement processes that are defective in those aneuploids. We therefore identified genes whose duplication compensates growth defects in *ssd1Δ* aneuploids under the conditions used here (Comparison 4 in Figs 2B and **5A**). There were 445 genes whose duplication was beneficial to at least one *ssd1Δ* aneuploid strain (FDR < 0.05) and that displayed a higher fitness score than observed in corresponding wild-type aneuploids and both euploids (see Methods and S1 Data). We identified the greatest number of beneficial genes in *ssd1Δ* cells with an extra copy of Chr12, possibly due to increased statistical power from 4 replicates (but perhaps also influenced by the rDNA locus on this chromosome [83]), followed by *ssd1Δ* Chr4 aneuploid cells. The fewest beneficial duplications were found in the *ssd1Δ* Chr7 aneuploid, which is the least dependent on Ssd1 of the aneuploids studied here. To validate, we tested 13 plasmids expressed in Chr12 aneuploids; 8 of the 13 genes significantly improved growth in the *ssd1Δ* aneuploid relative to empty vector, confirming library measurements (S3A Fig).

Comparing across strains revealed that many gene duplications were beneficial to multiple *ssd1Δ* sensitized aneuploids (**Fig 5B**, see Methods). We were especially interested in these genes, which may point to processes that are generally compromised upon chromosome duplication. 98 of these gene duplications showed positive fitness scores in at least 2 aneuploids, and 18 duplications were positive in at least 3 aneuploids. The group of 98 was enriched for genes encoding small GTPase-mediated signaling proteins (p = 3.7E-05, hypergeometric test) and mRNA processing proteins (p = 9.8E-04), while the group of 18 was enriched for translational regulators (p = 1.1E-03, hypergeometric test). Somewhat surprisingly, we did not find enrichment of genes linked to protein folding or the ubiquitin-proteasome system, which were previously associated with aneuploidy tolerance in the *ssd1-* W303 strain [45]. Of the five plasmids with positive fitness scores in all four aneuploid *ssd1Δ* strains, four encode proteins directly implicated in translational regulation: poly-A binding protein Pab1, translational repressor Sbp1, and eIF5a elongation factor Hyp2, which was cloned along with uncharacterized gene *UTR5* on two of the selected plasmids. The fifth gene, *GLC7*, encodes PP1 phosphatase that can act on translational regulator eIF2a in yeast [46], as well as on other proteins. We confirmed that *PAB1*, *SBP1*, and *HYP2/UTR5* all significantly improve growth rates of *ssd1Δ* Chr12 aneuploid cells relative to empty vector (p < 0.05, S3A Fig). We tested the *HYP2/UTR5* plasmid in other strains and found that it also benefited *ssd1Δ* with Chr4 duplication and, to a minor degree that just missed significance (p = 0.08), the *ssd1Δ* strain with Chr7 duplicated (S3 Fig).

We performed a similar analysis for the wild-type aneuploids, and identified 66 genes that provided a benefit to two or more wild-type aneuploids relative to batch-paired euploid (S1 Data). Interestingly, ten of these genes overlap the 98 genes that were beneficial to multiple *ssd1Δ* aneuploids (S4 Fig). One of these was poly-A binding protein encoded by *PAB1*—indeed, duplication of *PAB1* provided a mild but significant benefit to the tested wild-type YPS1009_Chr12 strain (S3A Fig).

### Deleterious gene duplicates are also involved in translation

In retrospect, we realized that several of the genes whose duplication was especially *deleterious* in *ssd1Δ* euploid and wild-type aneuploid cells are also connected to translation (**Fig 4**). Among those genes was *OAZ1*, which encodes an anti-enzyme that negatively regulates polyamine synthesis [47]. Polyamines are precursors of hypusine, a non-canonical amino acid required for Hyp2 function [47–48, reviewed in 49]. Oaz1 production is regulated via a programmed ribosomal frameshift as well as a programmed ribosome stall [47,50], which together serve as readouts of translational fidelity that is in part dependent on Hyp2 [51]. Overproduction of Oaz1 is expected to indirectly inhibit Hyp2 function by downregulating the synthesis of polyamines required for hypusination. Intriguingly, *OAZ1* is encoded on Chr16, whose duplication is essentially inviable in the *ssd1Δ* background. Several other genes from Fig 4B are also linked to these processes: *SAM3* involved in S-adenosylmethionine (SAM) and polyamine transport [52], *MTQ1*, a SAM-dependent translational regulator [53], and *RPG1*, a subunit of eIF3 with roles in translation reinitiation as well as stop codon readthrough [54,55]. Together, these links reinforce the idea that under the conditions studied here, *ssd1Δ* aneuploid strains and, to a lesser extent wild-type aneuploids, may have defects related to translation.

Our previous work showed that the Chr12 aneuploid and especially *ssd1Δ* Chr12 aneuploid without the drug-resistance cassette are sensitive to low doses of nourseothricin (NTC) [21], a translation-elongation inhibitor that at low doses stalls ribosomes, induces tRNA misincorporation and produces protein misfolding [56,57]. To test if this is a generalizable feature of aneuploid strains, we investigated other chromosome duplications for sensitivity to NTC. Indeed, all *SSD1+* aneuploids without the drug-resistance gene grew significantly slower than expected based on an additive genetic model of aneuploidy and NTC effects (p < 0.05, replicate-paired t-test, Fig 5C, see Methods) except the Chr7 aneuploid, which missed the significance cutoff. On the other end of the spectrum, duplication of the highly Ssd1-dependent Chr16 produced the greatest sensitivity to the drug. All of the *ssd1Δ* aneuploids treated with NTC grew reproducibly slower than expected across all replicates. Importantly, the *ssd1Δ* euploid shows no sensitivity to NTC, indicating a genetic interaction between *SSD1* deletion and extra chromosomes. Together, the sensitivity to NTC implicates translation as a particular vulnerability of wild-type aneuploids, which is exacerbated in the absence of *SSD1*.

One consideration is if the genes identified as beneficial in our study are related to residual effects of aminoglycoside antibiotics used in our library selection, even though strains carry the resistance genes. The library selection was done in the presence of G418 to maintain the plasmid library and nourseothricin to maintain the extra chromosome. It is possible that remaining sensitivity to NTC or G418 persists even when cells harbor the drug-resistance markers. To investigate, we performed several controls (S5 Fig). To the extent that we can detect, aneuploids with the G418 resistance marker did not show appreciable G418 sensitivity, with the exception of slightly reduced growth of the *ssd1Δ* Chr4 aneuploid that is already extremely slow growing. This was important, because validation of beneficial genes was done in the presence of G418 selection (without additional NTC). Furthermore, of two sets of strains tested (wild-type and *ssd1Δ* strains with extra Chr12 or Chr15), none of the strains with the NTC resistance cassette were sensitive to NTC under the conditions studied here. We also compared library results from this study to another recent study from our lab investigating the Chr12 aneuploid with the Moby 1.0 library grown in the absence of NTC (G418 was still required to maintain the library) [58]. Despite experimental differences, the results were highly similar between the two studies (S5C and S5D Fig), including the wild-type aneuploid sensitivity to the 218 genes shown in Fig 3B. We note that these genes were identified by their deleterious effects in the *ssd1Δ* euploid, which shows no sensitivity to aminoglycoside drugs. Finally, our validation experiments to test beneficial effects of individual plasmids (S4 Fig) was done in rich medium without NTC. Thus, while it remains possible that the beneficial genes identified here are influenced by unrecognized residual drug sensitivity of strains with the drug-resistance genes, our results together implicate a liability of aneuploid cells related to translation.

## Discussion

Although a rich body of literature describes phenotypes associated with chromosome duplication, the molecular causes of those phenotypes and aneuploidy toxicity in general remain poorly understood. Previous studies suggest that the products of amplified genes are to blame [4], but whether this is due to specific products and their associated properties or functions, or to a general burden associated with the increased genic load had been unknown. Many studies of Down syndrome (DS) focus on single genes amplified on human chromosome 21 [9,10], and the effects of these genes are often studied via duplication in isolation in euploid tissue culture. On the other hand, several lines of evidence instead implicate the generalized burden of aneuploidy, independent of which chromosome is amplified. For example, Bonney *et al*. [5] found that extra copies of a few dosage-sensitive genes expressed on each chromosome were not sufficient to explain reduced proliferation of aneuploids in a sensitized laboratory strain. Several other studies emphasize consequences of aneuploidy that are karyotype-independent [4,11,13,14,38,59–61]. Many perspectives suggest that it is some combination of the two models, where deleterious genes on each chromosome combine with increased sensitivity of aneuploid strains that have an increased underlying burden on cellular systems.

Our results provide evidence for an important and distinct model: that the generalized burden of chromosome duplication sensitizes cells to the duplication of genes that are not deleterious on their own but become so in the aneuploid context. This model posits a genetic interaction between generalized and gene-specific effects of chromosome duplication in *SSD1+* cells. We propose that the generalized effect of chromosome amplification mimics to some degree the vulnerability of *ssd1Δ* euploid cells to specific gene duplications. This hypothesis suggests that the physiological limitations of *ssd1Δ* cells, including those related to translation (and perhaps other things), are also at play in *SSD1+* aneuploids.

Several lines of evidence support this model. On the one hand, we found that the growth defects incurred by chromosome duplication, in both wild-type and *ssd1Δ* cells, can be partly explained by a subset of toxic gene duplicates. Importantly, this group does not represent the most problematic genes in the wild-type euploid. In fact, many of these genes are near-neutral when duplicated in the euploid. Instead, the cellular context created by *SSD1* deletion, or by chromosome amplification in *SSD1*+ cells, induces a specific vulnerability that renders these duplications toxic. It is possible that this sensitivity is amplified by the selection conditions used here (see S5 Fig). Nonetheless, we hypothesize that this vulnerability in aneuploid strains emerges from the cumulative effect of amplifying many genes at once [32]. Our model—that particular gene duplicates become problematic in the context of aneuploidy—has important implications for studies on aneuploidy toxicity, including the etiology of human aneuploidy syndromes. For example, many DS studies investigate the impact of human-chromosome 21 genes by duplicating them individually in euploid cell lines [62–66]. Our results raise caution about modeling aneuploid phenotypes in euploids without considering the cellular context of aneuploidy.

Despite these important insights, several unanswered questions remain. Why do certain gene duplicates become deleterious in the aneuploid context and what is the role of Ssd1 in managing them? Among the 218 gene duplications especially deleterious in *ssd1Δ* euploid cells, the only shared signature we identified is that many are shorter than other genes. Short transcripts associate more with “closed loop” factors involved in mRNA circularization, which facilitates faster translation reinitiation [67,68]. This could be related to translational effects discussed below. Notably, we did not find these Ssd1-dependent genes to be more highly expressed at the RNA or protein level, nor did we find overrepresentation of genes encoding protein complexes, proteins with many protein-protein interactions, or proteins with a high propensity for disorder (see Methods). Work from our lab and others [32,69] strongly suggest that genes encoding multi-subunit complexes are not toxic when merely duplicated, likely because cells have evolved mechanisms to manage their moderate increase [16,32,70,71]. Therefore, the harmful effects identified in this study likely arise from unrecognized attributes, necessitating more research to investigate further.

Additionally, the mechanism by which Ssd1 influences these genes’ toxicity, if not directly regulating their mRNAs, remains unclear. Ssd1 interacts physically with the ribosome and other proteins that regulate translation (including Pab1 identified above as beneficial to *ssd1Δ* aneuploids) [72,73]. It also directly binds hundreds of mRNAs through direct contact [24]. One possibility is that Ssd1 has a general role in translation at many more mRNAs than those bound through direct contact. But another possibility is that misregulation of transcripts bound directly by Ssd1 triggers secondary, widespread consequences on translation dynamics or fidelity, resulting in toxic effects at other duplicated genes. Precedence exists for this situation. For instance, Aviner *et al*. [74] demonstrated that the ribosome collisions on mutant Huntington transcript produce a cycle of dysfunction that sequesters eIF5a. This sequestration depletes eIF5a from other transcripts, altering translation of stress-responsive transcripts, in part by misregulating their upstream open reading frames (uORFs). Interestingly, Ssd1 has also been linked to transcripts with uORFs [75], although we found no enrichment for known uORFs among the 218 genes identified here. Nonetheless, this example demonstrates how altered regulation of Ssd1 targets could indirectly render a different set of gene duplicates toxic in that context.

We propose that aneuploidy creates a context in which translational efficiency and/or fidelity is perturbed. Several lines of evidence support this hypothesis. First, both the sensitized lab strain and wild strains lacking *SSD1* are highly sensitive to translation inhibitors including NTC—but only in the context of extra chromosomes [4,21,45]. This is consistent with a role for Ssd1 in translation, and suggests that Ssd1 is required to manage translational stress in aneuploid cells [26,30]. But wild-type aneuploids with functional *SSD1* (and lacking the NTC resistance gene) are also more sensitive to NTC than euploid strains [21], suggesting that a vulnerability to translational stress exists also in *SSD1+* cells. Studies in other organisms show that translational efficiency is also perturbed in response to monosomy, apparently driven by imbalanced expression of ribosomal proteins [76,77]. Thus other types of karyotype imbalance could also perturb global translation. Another line of evidence is that duplication of several genes involved in translation ameliorates the growth defect of *ssd1Δ* aneuploids, at least under the conditions used here. Finally, several recent studies from our lab (including those that use no aminoglycosides) identified other genes involved in translation that influence aneuploidy phenotypes, including genes linked to translational repression, P-body formation, and ribosome quality control [58,78]. Thus, mounting evidence points to a liability in translation in aneuploid yeast.

Several of these translational modifiers are implicated in tRNA misincorporation, which can lead to protein errors. An intriguing idea is that aneuploidy-induced translational stress provides the unrecognized source of proteostasis stress in aneuploid cells. Once again, Ssd1 may aid in generating hypotheses. Strains containing an *SSD1* hypomorph are more vulnerable to defects in the Elongator complex, which is essential for chemically modifying specific tRNA [29]. Lack of these modifications results in tRNA misincorporation, frameshifting, and increased protein aggregation [79–82]. That Ssd1 is linked to Elongator function and required for NTC tolerance (although only in the presence of extra chromosomes) suggests a role for Ssd1 in this process. We previously showed that NTC treatment of *SSD1+* aneuploids mimics signatures of protein aggregation found in *ssd1Δ* aneuploids [21]. Thus, exacerbating translational defects in aneuploid cells, through NTC treatment or *SSD1* deletion, may further overwhelm protein folding and maintenance systems leading to compromised proteostasis. Investigation of precisely how translational regulation intersects with proteostasis in aneuploids represents a fruitful area of ongoing inquiry.

## Materials and methods

### Strains and growth conditions

Further information about aneuploid panel, including strains and growth rates measured under standard conditions, can be found in [32]. All other strains used in this study are listed in S2 Table. In order to maintain aneuploidy over long-term growth, NATMX and His3MX6 were integrated by homologous recombination in the intergenic regions indicated in S2 Table, such that the two copies of the duplicated chromosome contained one or the other marker. Strains used in gene duplication experiments were transformed with a pool of the molecular barcoded yeast ORF library (MoBY 1.0) containing 5,037 barcoded CEN plasmids [40]. At least 25,000 transformants were scraped from agar plates for > fivefold replication of the library, and frozen glycerol stocks were made. Multiple independent transformations of the pooled library were performed for each strain (see competitive growth details below). Competitive growth was done in liquid synthetic media lacking histidine (SC-His) and with NTC (Werner BioAgents and Jena Bioscience, 100 mg/L) to maintain aneuploidy and G418 (Research Products International, 200 mg/L) for plasmid selection. We confirmed that most aneuploids are not sensitive to these drugs when they carry the drug-resistance cassette (S5 Fig). Experiments interrogating single genes were performed in rich YPD medium with G418 but in the absence of NTC in test tubes at 30°C with shaking. Strains to be compared were grown side-by-side in each replicate, allowing replicate-paired T-tests for statistical analysis. For NTC experiments in Fig 5C, cells were cultured in rich YPD medium for 3 hours into log phase; 1ug/mL NTC or an equivalent volume of water (vehicle control) were added; OD_600_ measurements were recorded beginning 1 hour after NTC addition. The observed growth rates for NTC-treated wild-type aneuploids was compared to the expected growth rates, calculated as the product of the no-NTC aneuploid growth rate and the % growth-rate reduction in the euploid treated with NTC. The observed growth rates for NTC-treated *ssd1Δ* aneuploids was compared to the expected growth rate, taken as the product of the % growth-rate reduction in the *ssd1Δ* aneuploid versus corresponding wild-type aneuploid in the absence of NTC treatment (the *ssd1Δ* effect) and the % growth-rate reduction in the NTC-treated wild-type aneuploid responding to NTC (the NTC effect).

### MoBY 1.0 competitive growth

Competition experiments were performed as in [40,83]. Briefly, 1 mL frozen glycerol stocks of library transformed cells were thawed into 100 mL of liquid SC-His with NTC (100 mg/L) and G418 (200 mg/L) at a starting OD_600_ of 0.05, then grown in shake flasks at 30°C with shaking. An aliquot of frozen stock represented the starting pool (generation 0) for each strain. After five generations, each pooled culture was diluted to an OD_600_ of 0.05 in fresh media to maintain cells in log phase. At 10 generations (5 generations where indicated) cells were harvested and cell pellets were stored at −80°C.

Data were collected in two batches. Batch 1 data were generated from two independent competitive growths from each of two independent transformations of the plasmid library, for a total of 4 replicates. Reproducibility of fitness scores was consistently high between independent growths from the same transformation stock (Pearson correlation coefficients PCC 0.87–0.96) and more modest across independent growths from different transformation stocks (PCC 0.44–0.67). Thus, to capture this variation, Batch 2 included three independent competition experiments, each from a different transformation of the pooled library. This ultimately yielded noisier fitness scores but greater confidence in reproducible effects. We pooled euploid controls from Batch 1 and Batch 2 for 7 replicates each of the wild-type euploid [32] and *ssd1Δ* euploid experiments. Fitness scores generated from Batch 1 and Batch 2 were reproducible, with PCC of 0.93 and 0.92 for wild-type euploid and *ssd1Δ* euploid, respectively.

### MoBY 1.0 sequencing

Plasmids were recovered from each pool using Zymoprep Yeast Plasmid Miniprep II (Zymo Research D2004-A). Plasmid barcodes were amplified using primers described in [84] and 20 PCR cycles. Barcode amplicons were pooled and purified using AxyPrep Mag beads (1.8X volume beads per sample volume) according to the manufacturer’s instructions (Axygen). Pooled amplicons were sequenced on one lane of an Illumina HiSeq 4000 to generate single-end 50 bp reads. Reads were mapped to 4,872 unique upstream tags (barcodes) [40]. This resulted in 126.2 million reads with exact matches to MoBY 1.0 barcodes in Batch 1 across 32 samples (average of ~3.9M reads/sample) and 154.7 million reads in Batch 2 across 51 samples (average of ~3.0M reads/sample). Sequencing data and barcode counts are available in the Gene Expression Omnibus (GEO) under accession GSE263221.

### MoBY 1.0 dataset analysis

To limit bias from barcodes that were under-represented in the starting pool, we removed barcodes in the bottom 5% of total counts at generation zero or with 0 reads across all samples (n = 303 for Batch 1 comparisons, n = 338 for Batch 2 comparisons, n = 313 for integrated comparisons across all 7 euploid replicates). A pseudocount of 1 was added to every measurement, and resulting data were normalized by TMM normalization [85] in edgeR version 3.36.0 [86]. TMM normalization produced very similar results to normalization by total barcodes sequenced. Barcodes that changed significantly in abundance in each experiment (i.e. after 10 generations of outgrowth compared to each starting pool) were identified using a glmQ function in edgeR with quasi-likelihood tests, using Benjamini and Hochberg multiple test correction and taking a false discovery rate (FDR) < 0.05 as significant [87]. A small number of genes cloned on multiple plasmids, and thus reported with multiple barcodes, were removed from downstream analysis. In total, barcodes representing 4,508 genes were analyzed across the two batches of experiments, interrogating indicated aneuploids and batch-paired euploids. Fitness scores correspond to the average log_2_(fold change) reported in edgeR output files. Hierarchical clustering was performed using Cluster 3.0 [88] and visualized using Java TreeView [89]. 5 generation collections (Chr4 *ssd1Δ*, Chr4 wild-type, and isogenic euploids) were analyzed separately. To maximize available statistical power, we combined and reanalyzed all wild-type and *ssd1Δ* euploid replicates from Batch 1 and Batch 2 (referred to as “integrated analysis”).

### Enrichments

Enrichments for GO terms were assessed using setRank version 1.0 [90] or GO Term Finder version 0.86 in the S*accharomyces* Genome Database [91], comparing to a background dataset of all measured genes. Statistical significance was assessed using Mann-Whitney U tests for continuous data and hypergeometric tests for categorical terms. DNA motif searches were performed on sequences -500 bp upstream and +100 bp downstream each gene’s open reading frame, using MEME suite [92] for novel motif discovery and SEA [93] to look for enrichment of genes whose upstream regions harbor previously identified motifs associated with Ssd1 binding, AKUCAUUCCUU and CNYUCNYU, respectively [22,24]. Propensity for disorder was quantified by % residues with IUPRED3 disorder propensity score >0.5 [94]. Ssd1 targets were compiled from [21,24]; the Bayne *et al*. targets comprise the 189 targets identified at 30 degrees C as at least 2-fold enriched at FDR < 0.05 [24]. Other mRNA/protein features as listed in [32] were analyzed as outlined in that study, along with age-dependent ribosome pause sites from [95,96]. None of those features aside of what is listed in the main text showed statistical significance.

### Modeling

Linear modeling was performed using ordinary least squares (OLS) regression as implemented in the statsmodels version 0.11.1 package for python3. Visual representations of the regressions were created using seaborn version 0.11.2, with 95% confidence interval plotted using the lmplot function. Predicted Ssd1 dependence (Figs 1 and 3D) or predicted aneuploid fitness relative to the euploid (Fig 4C) were taken as the sum of scores for genes indicated in each figure legend that are on each chromosome.

### Identifying detrimental/beneficial gene sets

Unless otherwise noted, detrimental/beneficial gene sets correspond to barcodes with statistically significant decreases/increases in abundance (log_2_(fold change) < 0 or > 0, respectively, and FDR < 0.05). The 218 genes described in the text were identified comparing 7 replicates each of wild-type and *ssd1Δ* euploid selections (FDR < 0.05). Genes more detrimental in wild-type aneuploid versus euploid were those with a negative log_2_ fitness score at FDR < 0.05 and a log_2_ fitness score difference > 1.5 as compared to wild-type euploid. To identify gene duplicates more beneficial in *ssd1Δ* aneuploids than other strains (Fig 5), we required a significantly beneficial effect (log_2_ fitness score> 0, FDR < 0.05) and a fitness score difference of at least 0.5 in log_2_ space compared to euploid wild-type, euploid *ssd1Δ*, and the corresponding wild-type aneuploid, if available. We used a lower magnitude threshold for this analysis since it was required in three separate comparisons. (The Dis4 *ssd1Δ* strain, which was collected at 5 generations, was compared to 5 generation collections of wild-type euploid, *ssd1Δ* euploid, and Dis4 wild-type aneuploid, respectively.) This identified 445 gene duplicates that met our criteria in at least one *ssd1Δ* aneuploid compared to other strains. We relaxed criteria to identify “commonly beneficial” overlapping genes shown in Fig 5B, still requiring a significantly beneficial effect (log_2_ fitness score > 0, FDR < 0.05) and a higher fitness score relative to comparison strains, but regardless of the magnitude of the difference. To identify genes especially beneficial in wild-type aneuploids, we required a significantly beneficial effect (log_2_ fitness score> 0, FDR < 0.05) and a fitness score difference of at least 0.5 in log_2_ space compared to wild-type euploid. We again relaxed this criterion to identify “commonly beneficial” genes, still requiring a significantly beneficial effect (log_2_ fitness score > 0, FDR < 0.05) and a higher fitness score relative to wild-type euploid, but regardless of the magnitude of the difference.

## Supporting information

S1 Fig Genes scored as neutral in the barcoded library are validated as neutral when strains are grown in isolation. Two genes that are confidently scored as neutral in the library experiment were selected for investigation. The figure shows the average and standard deviation of relative growth rates of each strain harboring the indicated MoBY 1.0 plasmid vs. empty vector (EV), grown in selective media (SC-His + NTC + G418), n > = 3. The experiment confirms that these plasmids are indeed neutral and validate the library normalization procedure applied in this study.
(TIF)

S2 Fig Linear modeling using fitness for a small handful of genes. As shown in Fig 3D but using only the top 10% most toxic genes in *ssd1Δ* euploid relative to wild-type euploid (x-axis).
(TIF)

S3 Fig Duplication of select plasmids improves growth of *ssd1Δ* aneuploids. A) Average and standard deviation of growth rates for the denoted MoBY 1.0 gene duplication plasmid versus empty vector (EV) in Chr12 aneuploids grown in rich medium (YPD + G418) without NTC (n > = 3, *PAB1*, *SBP1*, *HYP2/UTR5*; n = 2, *CDC48*, *CDC25*, *GCN20*, *RPS28B*, *YPT6*, *STM1*, *ARL1*, *ZDS1*, *ARF1*, *ARF2*). (*) indicates p < 0.05 of one-tailed, replicate-paired t-test between denoted strain and EV; (+) indicates p < 0.1. B) Average and standard deviation of growth rates for the *HYP2/UTR5* plasmid versus empty vector in the indicated strains grown in rich medium YPD + G418 without NTC (n> = 3); (*) indicates p < 0.05 from one-tailed, paired t-test comparing relative growth rates between *SSD1+* and *ssd1Δ* strains; (+) indicates p = 0.08. We were unable to maintain aneuploidy in this experiment in *ssd1Δ* cells with Chr15 duplication.
(TIF)

S4 Fig Overlap between genes beneficial to wild-type and *ssd1Δ* aneuploids. Venn diagram showing overlap of genes beneficial to two or more *ssd1Δ* aneuploids (n = 98) and those beneficial to two or more wild-type aneuploids (n = 66). 10 genes (displayed with common names) were identified in both analyses.
(TIF)

S5 Fig Aneuploid strains are not sensitive to aminoglycosides when they carry the resistance genes. One concern was that the sensitivity to low doses of NTC in *ssd1Δ* aneuploids lacking the drug-resistance gene persists in strains carrying drug resistance. We performed several controls to ensure that selection of aneuploidy or the plasmid library using NTC and G418 does not affect aneuploid growth in the library experiments. A) We generated YPS1009_Chr12 and _Chr15 aneuploids in which the two chromosomes were marked with *HIS3* and *URA3* (dark grey) instead of *HIS3* and *NATMX* (light grey) as used in our study. The growth rate of wild-type and *ssd1Δ* aneuploids with Chr12 or Chr15 duplication was indistinguishable (p>0.05, independent two-tailed t-test), showing that these strains are not sensitive to NTC when they carry the NATMX cassette (n = 3 except Chr12 *ssd1Δ* and Chr15 WT SC–His + NTC, where n = 2). B) We also compared growth of all four chromosome duplications, in wild-type and *ssd1Δ* cells, when cells carried an integrated copy of the KANMX cassette for G418 resistance (n = 3 except Chr12 *ssd1Δ* and Chr15 *ssd1Δ*, where n = 2). With the exception of *ssd1Δ* YPS1009_Chr4 (somewhat confounded by its extreme growth defect), none of the strains grew differently in YPD in the presence or absence of 200 mg/L G418 compared to control. (*) = p < 0.05, independent two-tailed t-test. C) We also compared our library results to a recent study done in the absence of NTC selection but using the same plasmid library [58]. Selection of the library requires G418, which is unavoidable in our study; however, comparing results afforded an opportunity to test the effect of NTC selection used in this study, even though the selection conditions were somewhat different (see [58] for details). Nonetheless, the agreement between the studies were very high considering genes called significant in our analysis (FDR < 0.05): the R^2^ between log_2_ fitness scores measured here versus in [58] that used no NTC is 0.78 for the euploid comparison (C) and 0.84 for the YPS1009_Chr12 aneuploid (D). Furthermore, of the 232 genes identified in the present study that are at least 2X more deleterious in YPS1009_Chr12 versus the euploid, 85.5% of them are also more deleterious in the YPS1009_Chr12 versus euploid grown in rich medium without NTC [58]. Among these are many of the genes shown in Fig 4B. Based on these controls, we conclude that the inherent sensitivity to NTC in aneuploid strains *that lack the drug-resistance marker* does not persist to an appreciable level in the majority of aneuploid strains that carry the drug resistance cassettes. Nonetheless, it remains possible that residual drug sensitivity in *ssd1Δ* aneuploids could enhance the beneficial effect of some genes identified in this study. We highlight that plasmid validation experiments (S3 Fig) were done in the absence of NTC, confirming that the impact of beneficial genes validated in this study cannot be explained by NTC sensitivity.
(TIF)

S1 Table Number of replicates and called genes for different experiments. logFC = log_2_(fold change) in barcode abundance, taken as fitness score. Cultures were grown for 10 generations unless otherwise noted.
(DOCX)

S2 Table Strains used in this study. (DOCX)

S1 Data Gene duplication fitness scores and associated gene lists. logFC: measured fitness score (log_2_(FC = fold change) in barcode abundance) in for the denoted strain. FDR: false discovery rate (B-H correction) corresponding to fitness score for the denoted strain. B1: data generated from Batch 1 growth experiments; B2: data generated from Batch 2 experiments; integrated_analysis: data generated from combined analysis of Batch 1 and Batch 2.
(XLSX)

S2 Data Data represented in Figs 1, 5C, S1, S3A, S3B, S5A and S5B. (XLSX)
