# Supplementary figures and images for: The response to single-gene duplication implicates translation as a key vulnerability in aneuploid yeast
Source: PLoS Genet. 2024 Oct 25;20(10):e1011454. doi: 10.1371/journal.pgen.1011454 (PMC11540229; doi:10.1371/journal.pgen.1011454)

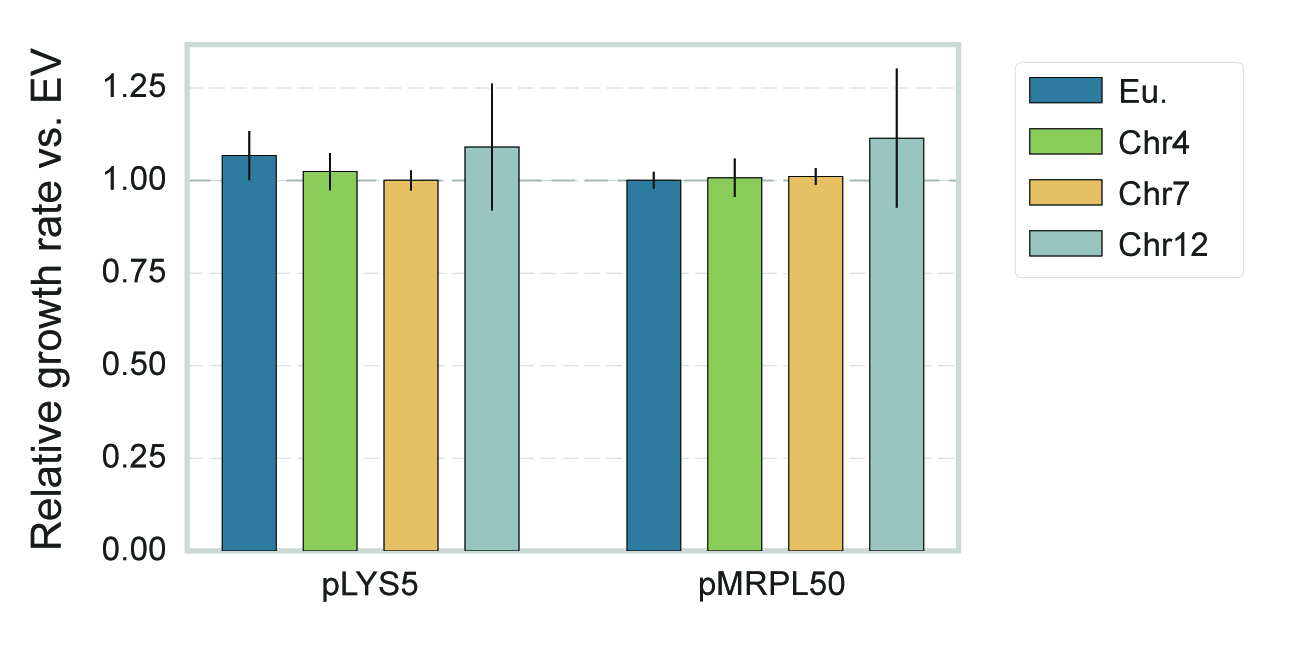

Supplement: S1 Fig — Two genes that are confidently scored as neutral in the library experiment were selected for investigation. The figure shows the average and standard deviation of relative growth rates of each strain harboring the indicated MoBY 1.0 plasmid vs. empty vector (EV), grown in selective media (SC-His + NTC + G418), n > = 3. The experiment confirms that these plasmids are indeed neutral and validate the library normalization procedure applied in this study. (TIF) [file pgen.1011454.s001.tif]

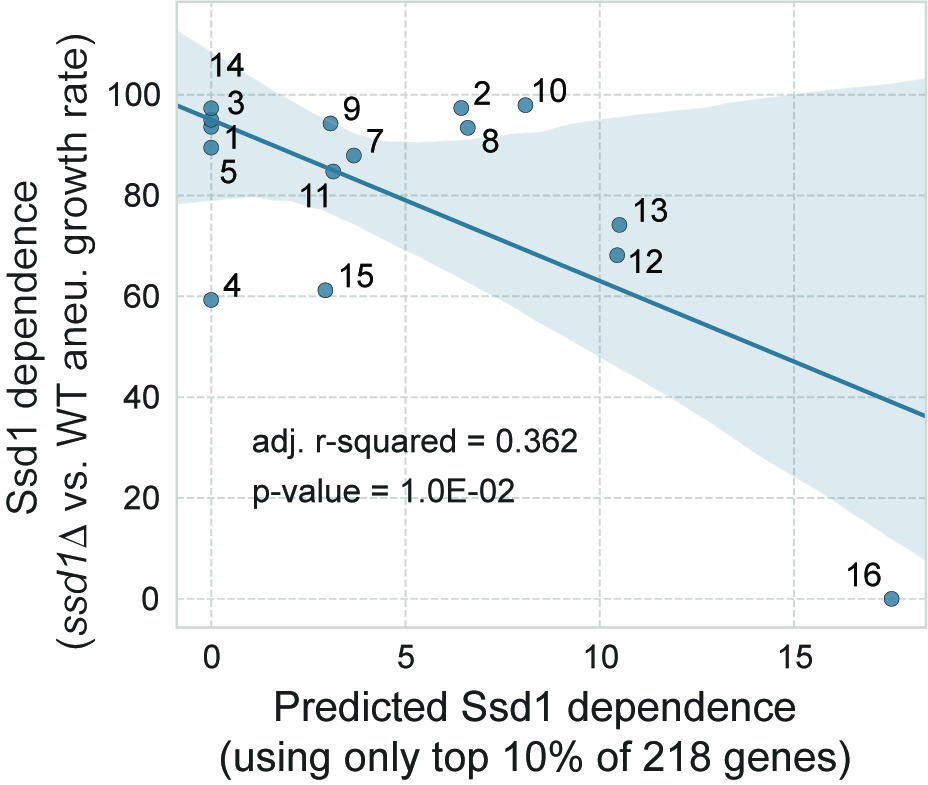

Supplement: S2 Fig — As shown in Fig 3D but using only the top 10% most toxic genes in ssd1Δ euploid relative to wild-type euploid (x-axis). (TIF) [file pgen.1011454.s002.tif]

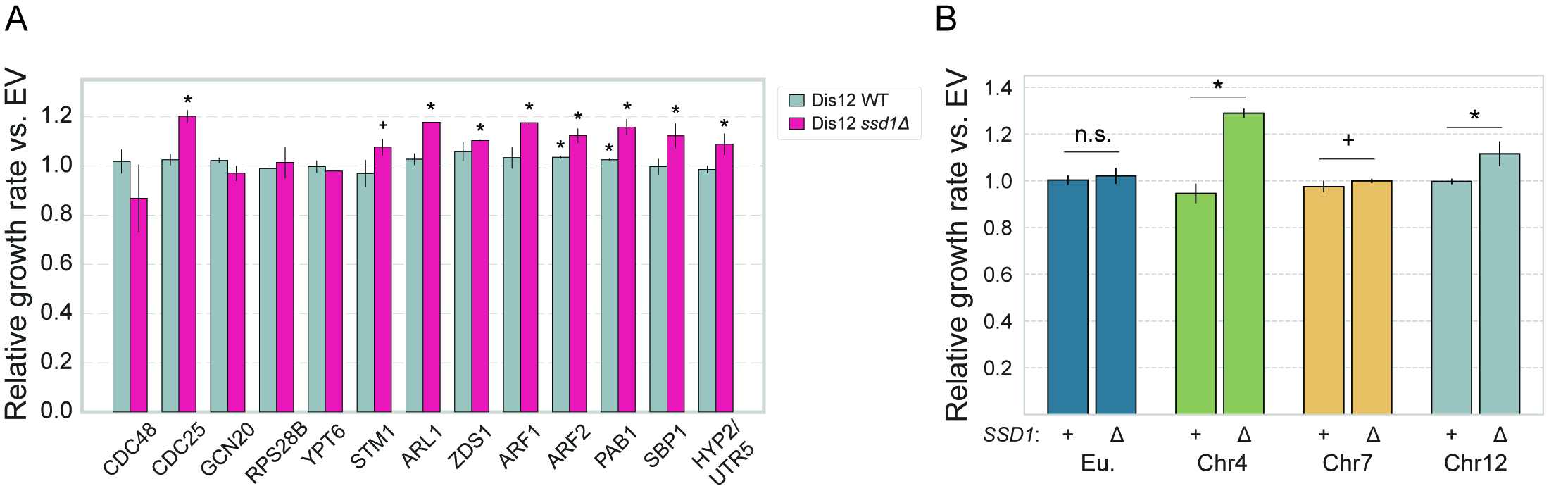

Supplement: S3 Fig — A) Average and standard deviation of growth rates for the denoted MoBY 1.0 gene duplication plasmid versus empty vector (EV) in Chr12 aneuploids grown in rich medium (YPD + G418) without NTC (n > = 3, PAB1, SBP1, HYP2/UTR5; n = 2, CDC48, CDC25, GCN20, RPS28B, YPT6, STM1, ARL1, ZDS1, ARF1, ARF2). (*) indicates p < 0.05 of one-tailed, replicate-paired t-test between denoted strain and EV; (+) indicates p < 0.1. B) Average and standard deviation of growth rates for the HYP2/UTR5 plasmid versus empty vector in the indicated strains grown in rich medium YPD + G418 without NTC (n> = 3); (*) indicates p < 0.05 from one-tailed, paired t-test comparing relative growth rates between SSD1+ and ssd1Δ strains; (+) indicates p = 0.08. We were unable to maintain aneuploidy in this experiment in ssd1Δ cells with Chr15 duplication. (TIF) [file pgen.1011454.s003.tif]

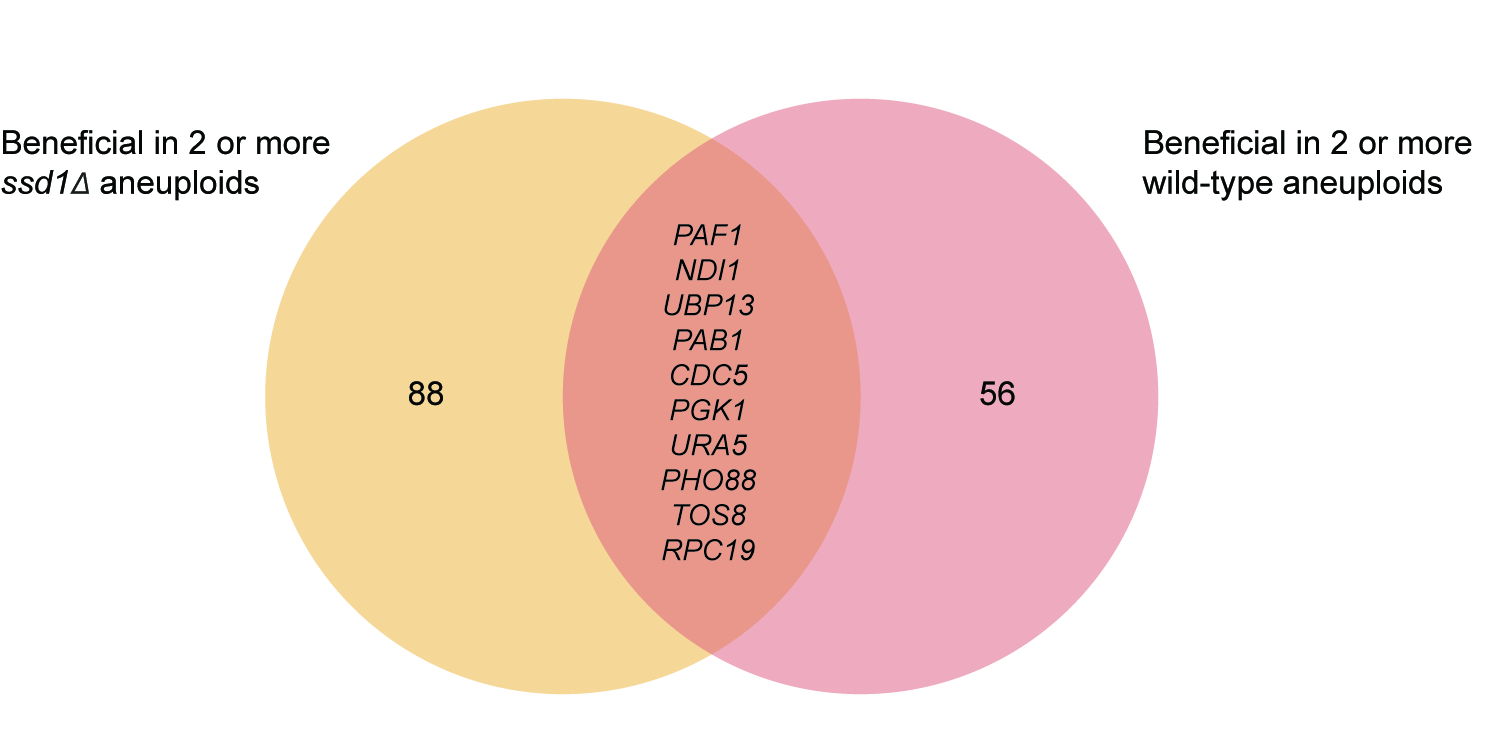

Supplement: S4 Fig — Venn diagram showing overlap of genes beneficial to two or more ssd1Δ aneuploids (n = 98) and those beneficial to two or more wild-type aneuploids (n = 66). 10 genes (displayed with common names) were identified in both analyses. (TIF) [file pgen.1011454.s004.tif]

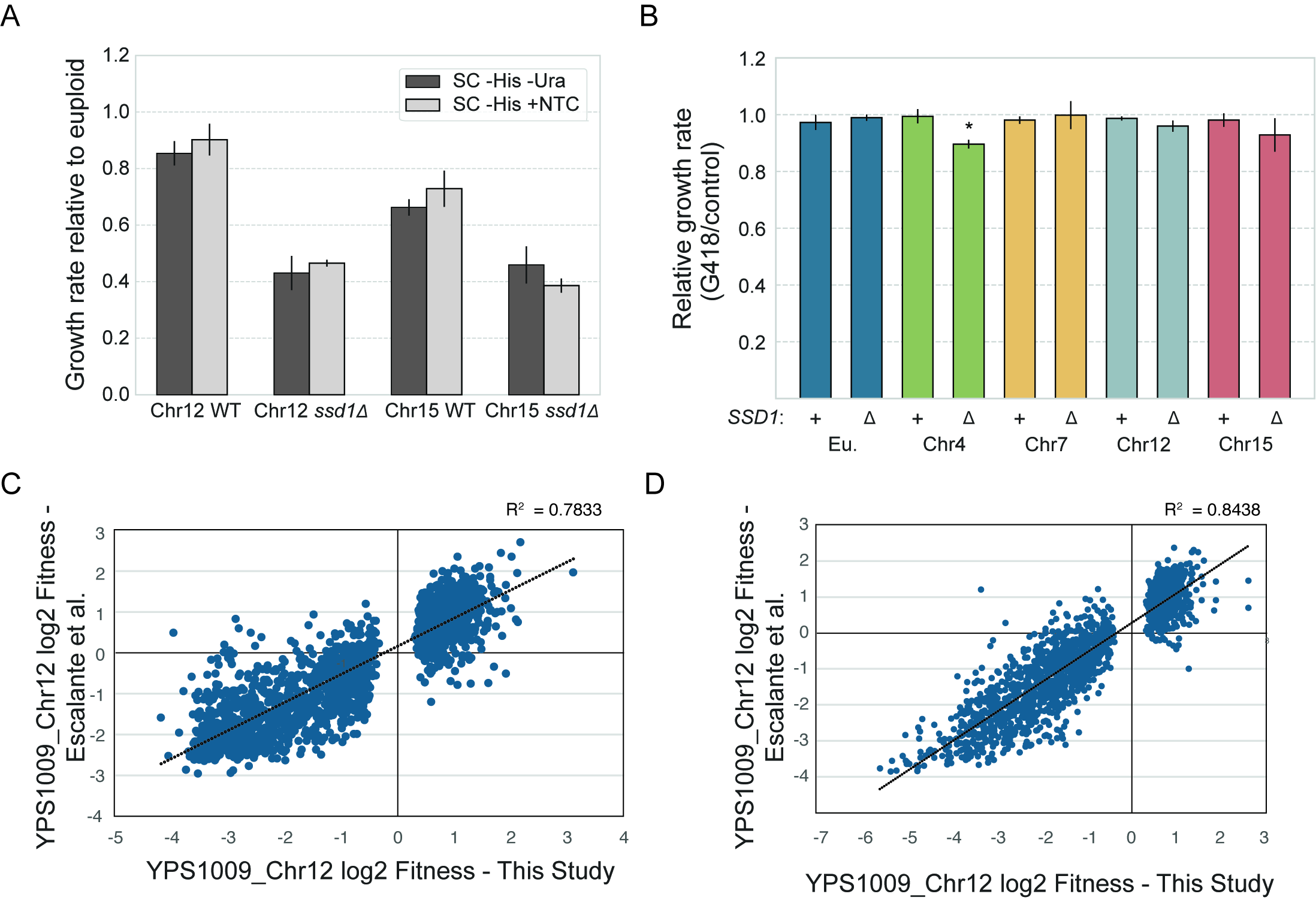

Supplement: S5 Fig — One concern was that the sensitivity to low doses of NTC in ssd1Δ aneuploids lacking the drug-resistance gene persists in strains carrying drug resistance. We performed several controls to ensure that selection of aneuploidy or the plasmid library using NTC and G418 does not affect aneuploid growth in the library experiments. A) We generated YPS1009_Chr12 and _Chr15 aneuploids in which the two chromosomes were marked with HIS3 and URA3 (dark grey) instead of HIS3 and NATMX (light grey) as used in our study. The growth rate of wild-type and ssd1Δ aneuploids with Chr12 or Chr15 duplication was indistinguishable (p>0.05, independent two-tailed t-test), showing that these strains are not sensitive to NTC when they carry the NATMX cassette (n = 3 except Chr12 ssd1Δ and Chr15 WT SC–His + NTC, where n = 2). B) We also compared growth of all four chromosome duplications, in wild-type and ssd1Δ cells, when cells carried an integrated copy of the KANMX cassette for G418 resistance (n = 3 except Chr12 ssd1Δ and Chr15 ssd1Δ, where n = 2). With the exception of ssd1Δ YPS1009_Chr4 (somewhat confounded by its extreme growth defect), none of the strains grew differently in YPD in the presence or absence of 200 mg/L G418 compared to control. (*) = p < 0.05, independent two-tailed t-test. C) We also compared our library results to a recent study done in the absence of NTC selection but using the same plasmid library [58]. Selection of the library requires G418, which is unavoidable in our study; however, comparing results afforded an opportunity to test the effect of NTC selection used in this study, even though the selection conditions were somewhat different (see [58] for details). Nonetheless, the agreement between the studies were very high considering genes called significant in our analysis (FDR < 0.05): the R2 between log2 fitness scores measured here versus in [58] that used no NTC is 0.78 for the euploid comparison (C) and 0.84 for the YPS1009_Chr12 aneuploid (D). Furthermor [file pgen.1011454.s005.tif]
