# Supplementary material for: The response to single-gene duplication implicates translation as a key vulnerability in aneuploid yeast
Source: PLoS Genet. 2024 Oct 25;20(10):e1011454. doi: 10.1371/journal.pgen.1011454 (PMC11540229; doi:10.1371/journal.pgen.1011454)
Supplement: S1 Table — logFC = log2(fold change) in barcode abundance, taken as fitness score. Cultures were grown for 10 generations unless otherwise noted. (DOCX) [file pgen.1011454.s006.docx]

|  |  | **Gene class (FDR < 0.05)** | |  |  |
| --- | --- | --- | --- | --- | --- |
| **Sample** | **# replicates** | **logFC > 0 (beneficial)** | **logFC < 0 (detrimental)** | **total** | **proportion detrimental** |
| Euploid WT Batch 1 (B1) | 4 | 899 | 1060 | 1959 | **0.54** |
| Euploid *ssd1Δ* Batch 1 (B1) | 4 | 709 | 912 | 1621 | **0.56** |
| Dis12 WT (B1) | 4 | 700 | 1132 | 1832 | **0.62** |
| Dis12 *ssd1Δ* (B1) | 4 | 972 | 1162 | 2134 | **0.54** |
| Euploid WT Batch 2 (B2) | 3 | 229 | 391 | 620 | **0.63** |
| Euploid *ssd1Δ* Batch 2 (B2) | 3 | 93 | 203 | 296 | **0.69** |
| Dis4 WT (B2) | 3 | 234 | 503 | 737 | **0.68** |
| Dis7 WT (B2) | 3 | 176 | 346 | 522 | **0.66** |
| Dis7 *ssd1Δ* (B2) | 3 | 130 | 442 | 572 | **0.77** |
| Dis15 *ssd1Δ* (B2) | 3 | 156 | 296 | 452 | **0.65** |
| Dis4 *ssd1Δ* (5 generations, B2) | 3 | 230 | 81 | 311 | **0.26** |
| Dis4 WT (5 generations, B2) | 3 | 565 | 674 | 1239 | **0.54** |
| Euploid WT (5 generations, B2) | 3 | 3 | 3 | 6 | **0.50** |
| Euploid *ssd1Δ* (5 generations, B2) | 3 | 44 | 59 | 103 | **0.57** |
| Euploid WT (B1 + B2 integrated analysis) | 7 | 1149 | 1254 | 2403 | **0.52** |
| Euploid *ssd1Δ* (B1 + B2 integrated analysis) | 7 | 886 | 1083 | 2011 | **0.55** |
|  |  |  |  |  |  |
