# Supplementary material for: The response to single-gene duplication implicates translation as a key vulnerability in aneuploid yeast
Source: PLoS Genet. 2024 Oct 25;20(10):e1011454. doi: 10.1371/journal.pgen.1011454 (PMC11540229; doi:10.1371/journal.pgen.1011454)
Supplement: S2 Table — (DOCX) [file pgen.1011454.s007.docx]

|  |
| --- |

S2 Table. Strains used in this study.

| Strain ID | Chromosome duplicated | Genotype | Reference |
| --- | --- | --- | --- |
| AGY1596 | - | h-YPS1009 Euploid MATa hoΔ1 | Rojas et al. 2024 [1] |
| AGY1611 | - | h-YPS1009 Euploid MATa hoΔ1 Chr12-intergenic region between YLR412W and YLR413W::NatMX | Rojas et al. 2024 [1] |
| AGY2141 | - | h-YPS1009 Euploid MATa ho::KanMX | This study |
| AGY1603 | - | h-YPS1009_Euploid MATa hoΔ1 ssd1Δ2 | This study |
| AGY1613 | - | h-YPS1009 Euploid MATa hoΔ1 ssd1Δ2 Chr12-intergenic region between YLR412W and YLR413W::NatMX | This study |
| AGY2142 | - | h-YPS1009 Euploid MATa ho::KanMX ssd1Δ2 | This study |
| AGY1835 | IV | h-YPS1009 Disome4 MATa hoΔ1 | Rojas et al. 2024 [1] |
| AGY1860 | IV | h-YPS1009 Disome4 MATa hoΔ1 his3-Δ Chr4-intergenic region between YDR032C and YDR033W::NAT/His3MX6 | This study |
| AGY1836 | IV | h-YPS1009 Disome4 MATa hoΔ1 ssd1Δ::KanMX/ssd1Δ::KanMX | Rojas et al. 2024 [1] |
| AGY1861 | IV | h-YPS1009 Disome4 MATa hoΔ1 his3-Δ Chr4-intergenic region between YDR032C and YDR033W::NatMX/His3MX6 | This study |
| AGY2143 | IV | h-YPS1009 Disome4 MATa hoΔ1/ho::KanMX | This study |
| AGY2144 | IV | h-YPS1009 Disome4 MATa hoΔ1/ho::KanMX ssd1Δ2/ssd1Δ2 | This study |
| AGY1843 | VII | h-YPS1009 Disome7 MATa hoΔ1 | Rojas et al. 2024 [1] |
| AGY1862 | VII | h-YPS1009 Disome7 MATa hoΔ1 his3-Δ Chr7-intergenic region between YGR249W and YGR250C::NatMX/His3MX6 | Rojas et al. 2024 [1] |
| AGY1844 | VII | h-YPS1009 Disome7 MATa ssd1Δ::KanMX hoΔ1 | Rojas et al. 2024 [1] |
| AGY1863 | VII | h-YPS1009 Disome7 MATa hoΔ1 his3-Δ ssd1Δ2 Chr7-intergenic region between YGR249W and YGR250C::NatMX/His3MX6 | Rojas et al. 2024 [1] |
| AGY2145 | VII | h-YPS1009 Disome7 MATa ho::KanMX | This study |
| AGY2146 | VII | h-YPS1009 Disome7 MATa ho::KanMX ssd1Δ2 | This study |
| AGY1598 | XII | h-YPS1009_Disome12 MATa hoΔ1 | This study |
| AGY1612 | XII | h-YPS1009 Disome12 MATa hoΔ1 his3-Δ Chr12-intergenic region between YLR412W and YLR413W::NatMX/His3MX6 | Rojas et al. 2024 [1] |
| AGY1604 | XII | h-YPS1009_Disome12 MATa hoΔ1 ssd1Δ2 | This study |
| AGY1614 | XII | h-YPS1009 Disome12 MATa hoΔ1 his3-Δ ssd1Δ2 Chr12-intergenic region between YLR412W and YLR413W::NatMX/His3MX6 | Rojas et al. 2024 [1] |
| AGY2137 | XII | h-YPS1009 Disome12 MATa hoΔ1 his3-Δ ura3-Δ Chr12-intergenic region between YLR412W and YLR413W::CaURA3/SpHIS3 | This study |
| AGY2138 | XII | h-YPS1009 Disome12 MATa hoΔ1 his3-Δ ura3-Δ Chr12-intergenic region between YLR412W and YLR413W::CaURA3/SpHIS3 ssd1Δ2 | This study |
| AGY2147 | XII | h-YPS1009 Disome12 MATa hoΔ::KanMX | This study |
| AGY2148 | XII | h-YPS1009 Disome12 MATa hoΔ::KanMX ssd1Δ2 | This study |
| AGY1839 | XV | h-YPS1009 Disome15 MATa hoΔ1 | Rojas et al. 2024 [1] |
| AGY1840 | XV | h-YPS1009 Disome15 MATa hoΔ1 ssd1Δ::KanMX | Rojas et al. 2024 [1] |
| AGY1859 | XV | h-YPS1009 Disome15 MATa, hoΔ1 HIS3/his3 Δ::NatMX ssd1Δ2 | This study |
| AGY2139 | XV | h-YPS1009 Disome15 MATa, hoΔ1 ura3-Δ HIS3/his3Δ::CaURA3 | This study |
| AGY2140 | XV | h-YPS1009 Disome15 MATa, hoΔ1 ura3-Δ HIS3/his3Δ::CaURA3 ssd1Δ2 | This study |
| AGY2149 | XV | h-YPS1009 Disome15 MATa hoΔ::KanMX | This study |
| AGY2150 | XV | h-YPS1009 Disome15 MATa hoΔ::KanMX ssd1Δ2 | This study |
| AGY1847 | XVI | h-YPS1009 Disome16 MATa hoΔ1 | Rojas et al. 2024 [1] |

**References**

1. Rojas J, Hose J, Dutcher HA, Place M, Wolters JF, Hittinger CT, et al. Comparative modeling reveals the molecular determinants of aneuploidy fitness cost in a wild yeast model. Cell Genomics. 2024;0: 100656. doi:10.1016/J.XGEN.2024.100656
